# Protocol for an economic evaluation alongside a natural experiment to evaluate the impact of later trading hours for bars and clubs in the night-time economy in Scotland: The ELEPHANT study

**DOI:** 10.1136/bmjopen-2024-095241

**Published:** 2025-05-14

**Authors:** Nurnabi Sheikh, Houra Haghpanahan, Jim Lewsey, Colin Angus, Carol Emslie, Niamh Fitzgerald, Emma McIntosh

**Affiliations:** 1 Health Economics and Health Technology Assessment (HEHTA), School of Health and Wellbeing, University of Glasgow, Glasgow, UK; 2 School of Medicine and Population Health, University of Sheffield, Sheffield, UK; 3 Research Centre for Health, School of Health and Life Sciences, Glasgow Caledonian University, Glasgow, UK; 4 Institute for Social Marketing and Health, University of Stirling, Glasgow, UK

**Keywords:** PUBLIC HEALTH, HEALTH ECONOMICS, Health policy

## Abstract

**ABSTRACT:**

**Introduction:**

The night-time economy comprises various sectors, including hospitality, transportation and entertainment, which generate substantial revenues and contribute to employment opportunities. Furthermore, the night-time economy provides spaces for leisure activities, cultural expression and social interaction. On-trade alcohol premises (places where consumers can buy and consume alcohol such as bars, pubs, clubs and restaurants) are a significant component of this night-time economy, functioning as focal points for socialising, entertainment and cultural events. However, when on-trade alcohol premises stay open later at night, this can be associated with negative public health impacts including increased alcohol consumption, intoxication, assaults, injuries and burden on public services including ambulance call outs, hospitalisations and increased impacts on criminal justice services. The evidence on the societal impact of policies to ‘later’ trading hours for bars and clubs in the night-time economy is limited. This protocol details the design of an economic evaluation of policy to later trading hours for bars and clubs in the night-time economy alongside the ELEPHANT study (National Institute for Health and Care Research (NIHR) Public Health Research, ref:129885).

**Methods and analysis:**

The research design is an economic evaluation alongside a natural experiment within the ELEPHANT study carried out in Glasgow and Aberdeen. The economic evaluation has been designed to identify, measure and value prospective resource impacts and outcomes to assess the costs and consequences of local policy changes regarding late night trading hours for bars and clubs. A number of economic evaluation frameworks will be employed. A cost-effectiveness analysis (CEA) is appropriate for assessing the effectiveness of complex interventions when the impacts of policy are measured in natural units. Therefore, a CEA will be conducted for the primary consequence, alcohol-related ambulance call-outs, using a health service sector perspective. Since this outcome is essentially a cost, the CEA will also be reported as a cost-analysis. A cost-consequence analysis will also be performed for the primary and secondary consequences including all ambulance call-outs and reported crimes to evaluate the full economic impacts of later trading hours for bars and clubs in the night-time economy. The analysis will be conducted from a wider societal perspective, including health sector, criminal justice system, business and third sector perspectives and will be in line with the recent National Institute for Health and Care Excellence guidance and recommendations.

**Ethics and dissemination:**

The economic evaluation of the ELEPHANT study will be conducted using secondary data. Thus, no ethical approval is required for this economic evaluation. However, ethical approval for the ELEPHANT study has been granted from the University of Stirling’s General Research Ethics Committee, and prior consent has also been obtained from the participants, if involved. The results of this study will be disseminated through peer-reviewed publications in journals and national and international conferences.

**STRENGTHS AND LIMITATIONS OF THIS STUDY**

- This is the first economic evaluation of a natural experiment to evaluate the impact of later trading hours for bars and clubs on alcohol-related ambulance call-outs and reported crimes in Scotland.
- A broad societal perspective, including health services, criminal justice system, business and a third sector perspective will be considered.
- This study will include a cost-effectiveness analysis, a cost-analysis and a cost-consequence analysis framework.
- This natural experiment nature of this study means that we cannot collect and incorporate all economic data such as benefits/gains to the night-time economy, alcohol-related road traffic accidents and productivity losses to individuals.

## Introduction

The night-time economy (NTE) refers to the economic activity that takes place during the evening and early morning hours between 18.00 and 6.00.^1^,^3^ The NTE comprises various sectors, including transportation, entertainment and hospitality, which generate substantial revenues and contribute to employment opportunities.^3 4^ The NTE provides spaces for leisure activities, cultural expression and social interaction. In 2019, 1.95 million people were employed in the NTE, which produced 5% of the overall gross domestic product (GDP) of the UK between 2011 and 2019.^5^ On-trade alcohol premises (places where consumers can buy and consume alcohol such as bars, pubs, clubs and restaurants) are a significant component of the NTE, functioning as focal points for socialising, entertainment and cultural events.^1^ Hence, in addition to contributing to revenue generation beyond the traditional 9-to-5 workday, alcohol consumption in the NTE is a popular pastime for many individuals and a growing aspect of night-time activities in urban environments.^2 4^ However, the proliferation of later trading hours for bars and clubs in the NTE can lead to excessive alcohol consumption and also raises concerns related to public health, safety and potential negative impacts including late night ambulance call-outs and increased crimes and assaults.^2^,^46^

Alcohol consumption is a major contributor to the burden of both communicable and non-communicable diseases globally,^12^,^15^ is the leading cause of preventable death in people aged 15–49 years, underpins increased crime, road traffic accidents and violence^13^,^16^ and imposes a huge health and economic burden.^12^,^17^ In 2016, harmful use of alcohol led to the premature deaths of three million people globally, accounting for 5.3% of all deaths, including 0.9 million injury-related deaths attributable to road injuries, self-harm and interpersonal violence.^12^ Furthermore, it is responsible for 132.6 million disability-adjusted life years (DALYs) annually, which accounts for 5.1% of the total global burden of disease and injury.^12^ An international systematic review and modelling study estimated the economic costs of harmful alcohol consumption to be an average of 2.6% of GDP in 2019.^18^ In England, alcohol-specific deaths were 7556 in 2021 and there were 342 795 alcohol-specific hospital admissions between 2021 and 2022.^19^ In 2004, 9.5% of presentations in the emergency department in Australia and New Zealand were found to be alcohol-associated.^20^ Between 2006 and 2014, the rate of alcohol-related emergency department visits in the USA increased from 1223 to 1802 per 100 000 people, resulting in a 272% increase in total costs from US$4.1 billion to US$15.3 billion.^21^ In Scotland, 1276 people died in 2022 from a cause wholly attributable to alcohol (alcohol-specific) with a 2% increase from 1245 in 2021; that is, an average of 25 people every week.^22^ In 2022/2023, 31 206 alcohol-related hospital admissions were recorded in Scotland; among them, 28 800 were admitted in general acute hospitals, which is equivalent to 532 hospital stays per 100 000 population and is about 3.4 times greater than the number of alcohol-related hospital stays recorded in 1981/1982.^23 24^

The harmful use of alcohol incurred an estimated cost of £3.56 billion (2007) for the Scottish economy, which is equivalent to £6.2 billion (inflation adjusted) in 2024; this translates to around £1756 per adult (adult: aged between 16 years and 65 years based on 2022 census data).^25^ Of this £3.56 billion (2007), health and social care expenses were estimated to account for £500 million (ie, inflation adjusted £869 million in 2024) a year.^25^ There is substantial evidence that reducing alcohol availability, regulating selling hours, increasing the price of alcohol and regulating marketing policy can prevent alcohol-related harms.^26^,^29^ In contrast, late-night alcohol sales are associated with increased rates of crimes, injuries, disorder and use of emergency services including accident and emergency (A&E) services, criminal justice system services, ambulance services and fire services. In the northeast of England from April 2009 to March 2010, 10% of ambulance call-outs were alcohol-related through a manual check of electronic records of northeast ambulance service, with a peak concentration between midnight and 1.00, largely on Friday and Saturday nights.^30^ Using a novel free-text algorithm, 16% of ambulance call-outs were found to be associated with alcohol-related incidents in Scotland.^31^ However, alcohol-related ambulance call-outs increased to 18.5% on weekends and further to 28.0% on weekend nights (from 18.00 to 6.00).^31^ In addition, they incurred an estimated £31.5 million costs in 2019 based on the average cost of an ambulance call-out.^31 32^ In 2019/2020, in Scotland, 44% of violent crimes involved offenders who were under the influence of alcohol; further, 20% of victims reported drinking alcohol immediately prior to the incidents.^33^ The annual cost of assaults in Scotland is estimated at >£1.5 billion despite progress made in tackling violence.^34^

On weekends in Scotland in 2019, the highest concentration of alcohol-related ambulance call-outs occurred between 21.00 and 1.00, whereas the highest concentration of non-alcohol-related call-outs appeared between 10.00 and 21.00; revealing that alcohol-related ambulance call-outs are more prevalent after midnight.^31^ Systematic reviews show that extensions in late night opening of alcohol premises are associated with increased intoxication, assaults, injuries and burden on public services.^7^,^11^ An extra 1-hour extension to opening times of alcohol outlets in 18 Norwegian cities was associated with a 16% increase in violent crimes from 22.00 to 5.00^35^ and similarly was associated with 34% more alcohol-related ambulance call-outs from 2.00 to 6.00 in Amsterdam, Netherlands.^36^ Opening hours changed from 22.00 to 23.00 in Scotland in the ‘70s, and women were found (in a before/after survey) to have slightly increased consumption and a slight shift in drinking at later times.^37^

Economic evaluation is a useful tool for informing decision making and public sector resource allocation.^38^ Previous systematic reviews of economic evaluation of alcohol prevention policies show that reducing/restricting alcohol selling hours and days reduced alcohol consumption and is likely to be cost-effective in preventing DALYs.^39^,^41^ However, a review article on the methodological challenges of economic evaluation for alcohol prevention policies identified major methodological limitations in the available literature including lack of consideration of multisectoral and long-term outcomes, lack of wider societal perspective and limited use of recommended economic evaluation methods, for example, cost-consequence analysis (CCA) and cost-benefit analysis.^42^ Restricted retail hours and days are estimated to have contributed to the avoidance of 1009 DALYs in Estonia,^43^ 2163 DALYs in Denmark^44^ and 2700 DALYs in Australia.^45^ These studies, however, also reported limitations including quality of data, lack of evidence of intervention effects and being restricted to a health sector perspective.^43^,^45^ Hence, this study aims to conduct an economic evaluation from a wider societal perspective, with a more robust method to estimate policy effect.

No previous study in the UK has considered the impact of later trading hours for bars and clubs on alcohol-related ambulance call-outs, and there have been no studies in Scotland since the ‘70s.^37^ Moreover, no studies have evaluated the economic impact of later trading hours for bars and clubs in Scotland from the viewpoint of the health service or taken a wider societal perspective which considers impacts on the judicial sector and retail. Economic evaluations using natural experiment methodology based on routine data are becoming increasingly popular.^46^,^48^ Although randomised controlled trials (RCTs) have been recognised as the ‘gold standard’ for assessing causal effects, natural experiments (in which the effects of a naturally occurring event or phenomenon are being observed) are identified as a practical solution for evaluating policies where randomisation is often not possible and sometimes unethical.^40 49^ However, there is still a dearth of literature reporting economic evaluations alongside natural experiments. In addition, the existing literature on economic evaluation alongside RCTs does not focus on important methodological aspects for conducting economic evaluations alongside natural experiments, particularly those related to study design.^48^ For example, it provides limited guidance on addressing biases and confounding effects raised from a lack of randomisation, selecting appropriate comparators and managing routine data from multiple sources. In proposing an economic evaluation of two policy changes alongside a natural experiment in Aberdeen and Glasgow cities, this protocol addresses an important gap in the literature.

### Objective

The aim of this economic evaluation alongside a natural experiment is to evaluate the economic impact of two policy changes in Aberdeen and Glasgow. Economic evaluation includes a cost-effectiveness analysis (CEA) for the primary outcome (alcohol-related ambulance call-outs) from a healthcare perspective and a cost analysis transforming the primary outcome into a cost (alcohol-related ambulance call-outs) from a healthcare perspective. A CCA is also reported for the primary and secondary outcomes (all ambulance call-outs and reported crimes) from a broader societal perspective. Outcomes will be reported in terms of ‘consequences’, in line with a CCA framework recommended by the National Institute for Health and Care Excellence for reporting public health economic evaluations.^50^

## Methods and analysis

### The policy changes: later trading hours for bars and clubs in Glasgow and Aberdeen

In Glasgow, as of 12 April 2019, under a scheme planned by the Glasgow Licensing Board (GLB), 10 nightclubs among the 17 applications submitted (among 1350 on-premises outlets) were permitted to implement a variation in their licence, enabling them to open for an extra hour until 4.00 for at least 12 months. The scheme was described by the Licensing Board as an ‘opportunity to reward and continue to encourage great practice in the nightclub trade’, as the extra hour was only granted to premises meeting certain criteria. The licence holder had to demonstrate, to the satisfaction of the GLB, not only that the premises made a positive contribution to the late-night economy, but that they invested in safety and security measures for both staff and customers and promoted the licensing objectives. The scheme was subsequently expanded before and after the COVID-19 pandemic when all nightclubs/bars were closed by public health measures.

In Aberdeen, the local licensing board changed their policy to remove a requirement for late-night venues to provide ‘significant entertainment’, following a period when the term had been very broadly interpreted. The changes took place from 2017 such that, initially, premises that provided ‘significant entertainment’, and later any premises, were allowed to request/apply to stay open later as a result of changes in the new statement of licensing policy. From November 2018, bars and pubs (which previously had to close at various times up to 1.00) applied to expand their hours, some to 2.00 and some to 3.00, and these extensions were granted. Existing nightclubs in Aberdeen had already been permitted to open to 3.00. To date, approximately 38 bars/pubs (among 430 on-premises outlets) have been granted the later trading hours, bringing them closer to or up to the same closing time as nightclubs. Although both policy changes involve later trading hours for bars and clubs, the scope and level of exposure in Aberdeen, measured by the number of premises and the hours permitted, is greater than in Glasgow.

### Setting and location

In this natural experiment, the selection of target population and study location is constrained by the location of policy changes/implementation.^48^ This study includes two Scottish cities as intervention sites (*the estimated effect will be at city level*), where 10 licensed alcohol premises in Glasgow and 38 licensed alcohol premises in Aberdeen were permitted to operate with later trading hours. So, our study target population will be those residents in these cities. The outcomes in these intervention cities will be compared with concurrent control areas to assess the impact of later trading hours. Control areas will be selected from other Scottish cities where such policies are not in place. Additionally, we will generate a synthetic control for both Aberdeen and Glasgow. The control selection procedure and synthetic control methodology are described below. To develop a controlled before-and-after evaluation, we will consider the dates from which the later closing time was permitted to be used by premises.

### Study design

The ELEPHANT study, a mixed-method natural experiment, was designed to understand and evaluate the contribution of changes in trading hours for bars and clubs in Glasgow and Aberdeen to harms, services and costs in the local NTE and the implications for other major UK cities. Two different policy changes (described above) have led to later trading hours, which form the basis of the natural experiment. Hence, an interrupted time series (ITS) design will be used to compare the impact of alcohol policy changes between intervention and suitable control areas. ITS design is a widely used method for strengthening before-after study designs.^34^ A clear differentiation is required between preintervention and postintervention periods for an ITS study. The ‘time’ (date) of alcohol policy changes will be regarded as an interruption in the time series and will define preintervention and postintervention periods. Data will be collected before and after that interruption, and at least three variables, such as a time variable, a policy variable to indicate preintervention and postintervention periods and the outcome variable with values for each time point are required for an ITS study. A short-term outcome is also suggested for an ITS study. In our case, alcohol-related ambulance callouts and reported crimes are immediate effects of policy changes. The interruptions in Aberdeen occurred gradually following the approval date for the later trading hours of the 38 bars and clubs. ITS is one of the effective research designs to establish causality when RCTs are not practical.^51 52^ It permits statistical inference to be carried out to examine seasonality, autocorrelation, random fluctuations and heteroskedasticity, all of which might lead to bias in an assessment of the impacts of the interruption/intervention.^53 54^ In addition, we will use location-based control groups.^55^ Control groups which have not been exposed to the intervention under study have the potential to improve internal validity and will lead to a controlled ITS design.^56^ Further information on the process of control selection is described in the following section and is also published in the preprint statistical analysis plan.^57^ The economic evaluation frameworks employed alongside this design are outlined below.

### Overview of the economic analysis and logic model

Alongside the natural experiment design, we will conduct a CEA for the primary outcome of alcohol-related ambulance call-outs. This CEA will also revert to a cost analysis by monetising the alcohol-related ambulance call-outs. The policy of later trading hours for bars and clubs is considered a detrimental policy from a public health perspective, meaning the policy is expected to be associated with additional costs along with an expected increase in alcohol-related ambulance call-outs. The results of the CEA (and associated, monetised cost analysis) will be reported in terms of the incremental cost (additional cost required to implement the policy) associated with the incremental alcohol-related ambulance call-outs caused by the later trading hours, if any. In addition, a CCA will also be conducted in line with recent guidance, ‘*A Framework for conducting economic evaluations alongside natural experiments’*, to evaluate the economic impacts of later trading hours for bars and clubs in the NTE.^48^ Indeed, this is essentially a cost analysis, but for decision-making purposes, we will further deconstruct this into a CCA by reporting multisectoral costs and consequences. CCAs have been recommended for complex interventions that have multiple consequences and that are difficult to measure in a common unit. It includes all types of consequences, including health, non-health, negative and positive effects. It aims to give decision makers a comprehensive summary of the different costs and consequences, so a wider societal perspective will be taken to conduct the CCA to capture all possible costs and consequences that are feasible to measure. The CCA will be performed from the healthcare system, the criminal justice system and the wider societal perspective. Within the CCA framework, we will also consider retailer, social service and third sector perspectives by drawing evidence from the qualitative studies of the ELEPHANT study (see the interview topic guides for a qualitative study in online supplemental appendix 1). The CCA will assess the consequences of later trading hours for bars and clubs by measuring incremental alcohol-related ambulance call-outs and reported crimes in natural units. We will then identify, measure and value the associated resource costs across multiple sectors, including health sector, criminal justice system, business sector and third sector. The health sector will cover ambulance service costs, A&E service costs and inpatient care costs. The criminal justice system will account for police service costs, justice system costs and the costs of physical and emotional harm. The retail or business sector costs will include application processing costs, venue operating costs and staff salaries for the later trading hours. The third sector will include costs associated with the voluntary organisations such as street pastor, taxi marshal, etc. We will report the monetised values of incremental costs corresponding to these negative consequences across sectors. We cannot measure intangible costs/benefits (eg, consumer pleasure/losses) within the scope of our study. However, qualitative evidence on the retailer benefits (eg, additional profits/loss) and consumer benefits will be reflected in our discussion.

**Figure 1 F1:**
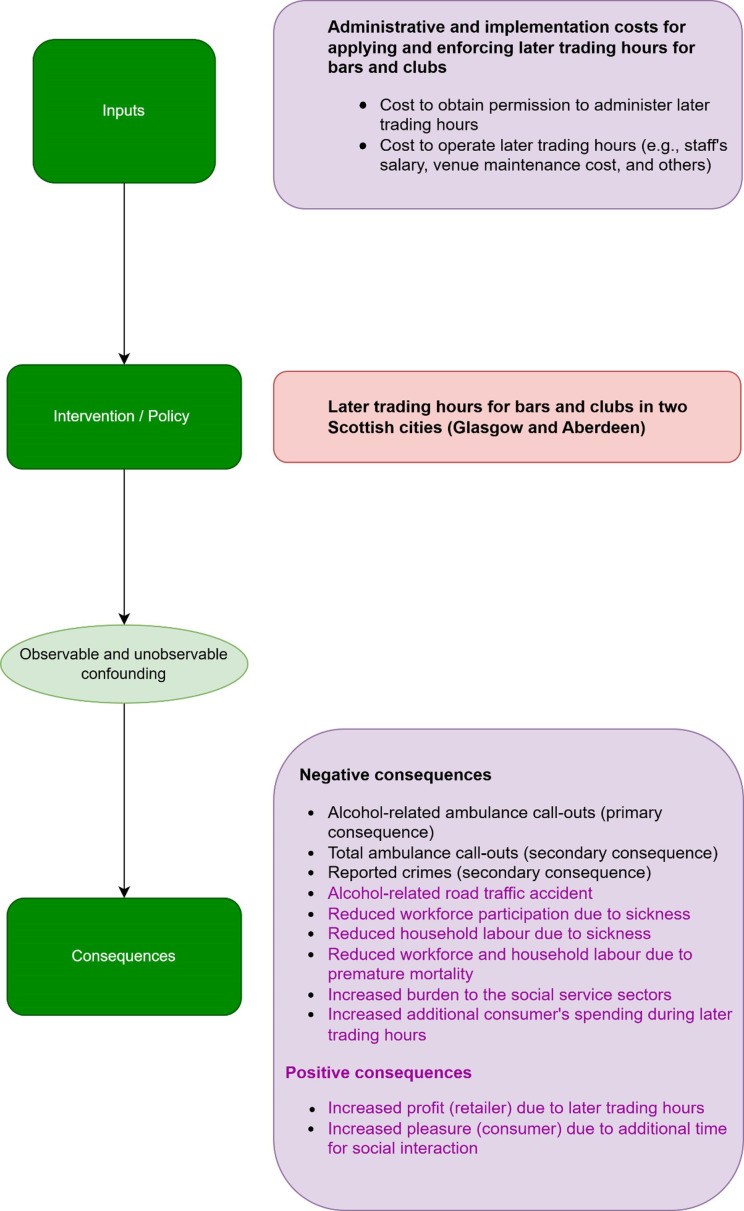
Economic evaluation logic model (data are not available for the purple-coloured inputs).

The economic evaluation will be conducted separately for the Aberdeen and Glasgow cities. An economic evaluation logic model has been developed (figure 1), building on the main study systems map. To conduct this complex economic evaluation, we have identified all possible resource uses and consequences, and our logic model (a conceptual framework) illustrates the causal pathways and interconnected linkages between these resources and consequences.^58^

However, it may not be possible to assess all potential resources and consequences in this economic evaluation, illustrated in an economic evaluation logic model. In light of our available data and sources assessment, we report in table 1 the resource uses/costs and consequences that might be amenable to assessment in our analysis from the logic model.

**Table 1:** Resources and outcomes with data sources that will be captured in the economic evaluation

Resource uses and outcomes	Data captured?	Data sources
**Resource uses/inputs**		
***Administrative and implementation costs for applying and enforcing later trading hours***		
Cost to obtain permission to administer later trading hours	Yes	Data from the qualitative studies and secondary published sources will be used.
Cost to operate later trading hours (eg, staff’s salary, venue maintenance cost and others)	Yes	Data from the qualitative studies and secondary published sources will be used.
**Outcomes/consequences**		
***Negative consequences***		
Alcohol-related ambulance call-outs (primary consequences)	Yes	Scottish Ambulance Service (time series data)
Total ambulance call-outs (secondary outcomes)	Yes	Scottish Ambulance Service (time series data)
Reported crimes (secondary outcomes)	Yes	Police Scotland (time series data)
Alcohol-related road traffic accident	No	Design not amenable to data capture.
Reduced workforce participation due to sickness	No	Design not amenable to data capture.
Reduced household labour due to sickness	No	Design not amenable to data capture.
Reduced workforce and household labour due to premature mortality	No	Design not amenable to data capture.
Increased burden to the social service sectors	Yes	Data from the qualitative studies and secondary published sources will be used.
Increased additional consumer’s spending during later or expanded premises hours	No	Design not amenable to data capture
***Positive consequences***		
Increased profit (retailer) due to later trading hours	No	
Increased pleasure (consumer) due to additional time for social interaction	No	

### Comparators

**Figure 2:**
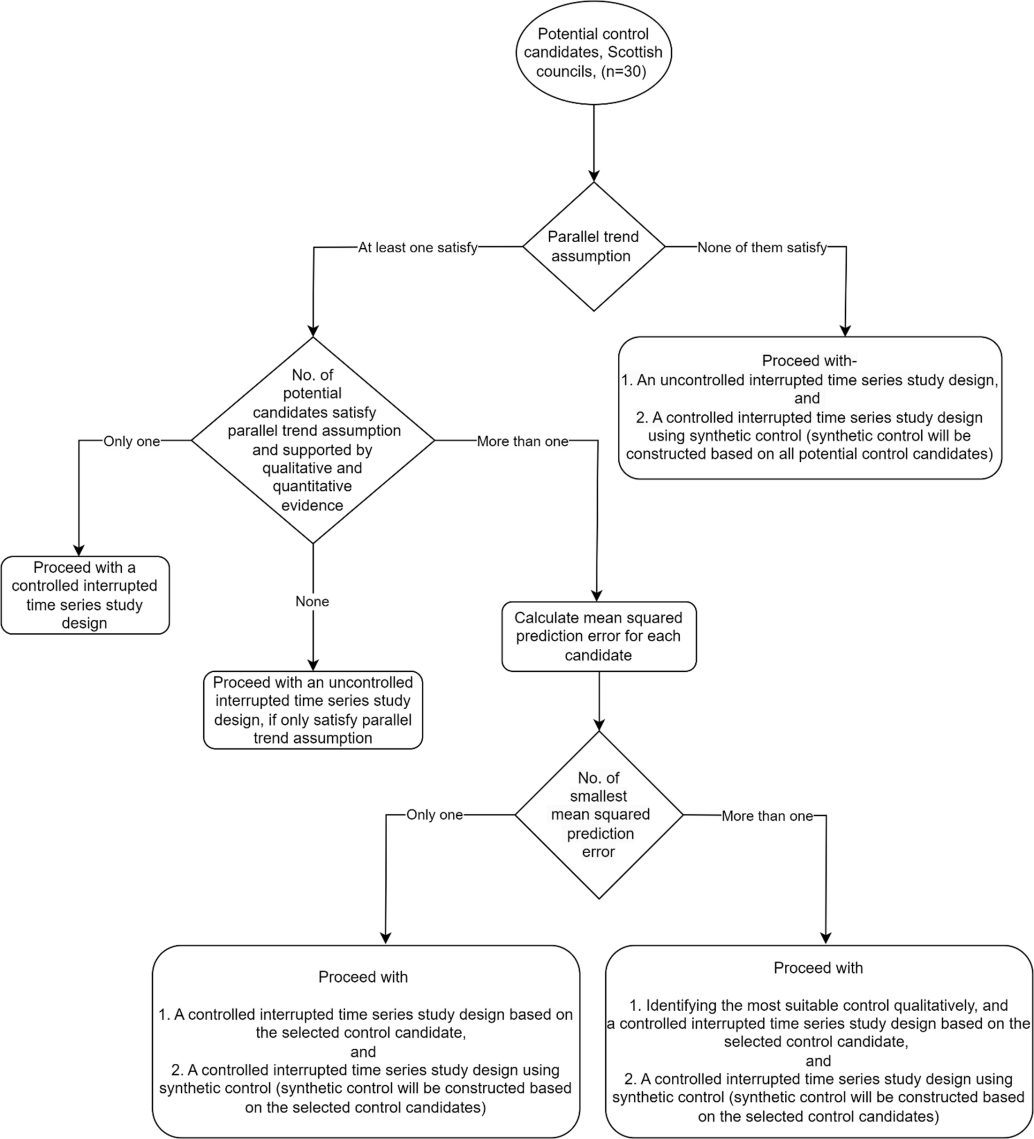
Flow diagram for the control selection procedure.

This study examines the impact of policy changes on outcomes and costs that are sensitive to both observed and unobserved confounding effects, due to the non-randomised study design. Therefore, based on the analysis of effectiveness, we will implement a control ITS study design (if we find a suitable control group). Economic evaluation methods also need to adapt to the challenges associated with non-randomisation and select an appropriate control group that closely approximates the intervention cities (Glasgow and Aberdeen). The objective of the control in an ITS study is to minimise biases caused by time-varying confounders, seasonality or other events that occurred during the implementation of policy changes.^52^ In this study, we will use a location-based control if we can identify any appropriate candidates. In a location-based control, different geographical areas with similar features, where the policy or intervention has not been adopted, are typically chosen. The process of suitable control selection and corresponding study design has been outlined in the flow diagram (figure 2).

In this study, we will assess the appropriateness of the 30 Scottish councils/cities (except intervention cities—Aberdeen and Glasgow) as potential controls. No policy changes that were comparable to those in Aberdeen and Glasgow took place during our initial assessment. Later trading hours were temporarily changed for holidays (eg, Christmas and New Year), but we expect that intervention cities were similarly impacted. The appropriateness of the potential control candidates will be tested by visually and statistically assessing the assumption of parallel trends.^59^ Graphically, we will observe the preintervention differences between the intervention and control groups. If the parallel test assumption holds, then the preintervention differences will be constant over time. There are no formal statistical tests for this assumption, and passing the parallel test does not necessarily mean that the parallel trend is true.^59^ However, failing the parallel tests will make the parallel trends assumption less plausible.

If none of the potential control candidates satisfy the parallel trends assumption, we will proceed with an uncontrolled ITS study design (figure 2). In addition, we will construct a synthetic control based on all potential control candidates since synthetic control does not need to satisfy parallel trends and will proceed with a controlled ITS study design.^60^ The synthetic control method (SCM) is widely recommended for ITS designs where multiple prospective control groups are available.^52 61^ The SCM is a data-driven process that builds a control series by calculating a weighted average of potential controls.^61^ To generate weights for the SCM, we will collect data from all Scottish councils/cities in addition to Glasgow and Aberdeen. The weights will be generated based on pre-intervention outcomes of potential control groups so that the preintervention trend of synthetic control matches well with the intervention. This process will be followed twice to generate synthetic controls for Aberdeen and Glasgow. Along with preintervention outcomes, other covariates representing time-varying confounding such as per capita gross disposable household income,^62^ number of alcohol premises/population-adjusted alcohol premises^63^ and weather (mean temperature and rainfall from the Centre for Environmental Data Analysis archive) will be used to generate weights for the synthetic control. In order to determine more accurate weights for the synthetic control series, we will incorporate population size-adjusted alcohol-related ambulance call-out and reported crimes rates for all regions in Scotland. We anticipate that, by using population-adjusted rates, the SCM series will yield a closer match to the observed outcomes prior to the intervention.

If at least one candidate satisfies the parallel trends assumption, we will assess the qualitative and quantitative evidence on control group suitability. For example, we will qualitatively assess whether other policy changes similar to later hours occurred at the selected control cities after our initial assessment through reviewing published reports, which might limit their suitability. In addition, we will consider on-premises outlet density per 1000 population, socioeconomic and geographical features to match with the intervention context. If potential controls only satisfy the parallel trends assumption but did not get support from the qualitative and quantitative assessment, we will proceed with an uncontrolled ITS study design. However, if only one candidate satisfies both parallel trends assumption and is supported by further evidence, we will proceed with a controlled ITS study design. We will calculate mean squared prediction errors (MSPEs) for each of them, if more than one candidate satisfies both the parallel trends assumption and has favourable qualitative and quantitative evidence support. If we get only one smallest MSPE with a clear differential to other values, we will use that corresponding control and proceed with a controlled ITS study design. In addition, we will construct a synthetic control based on the selected control groups and will proceed with a controlled ITS study design based on the synthetic control. However, if there are more than one similar smallest MSPEs (ie, two or more very close MSPEs), we will select the most suitable control qualitatively and will proceed with a controlled ITS study design based on the corresponding control. We will also construct synthetic control by incorporating selected control candidates and proceed only with a controlled ITS study design.

We will assess whether any such preintervention and postintervention changes are likely to be caused by the intervention, by analysing the data for the suitable control (if any). We will use a difference-in-difference approach, such that differences between intervention and control will be used as outcome variables for both primary and secondary outcomes.

### Resource use/costs

The cost components for this complex natural experiment involve integrating multiple NTE components, from differing perspectives, that could be affected by the policy changes in Aberdeen and Glasgow. There are multiple resource use components that we will endeavour to consider in costing. The costs related to the implementation of policy changes are considered from the retailer perspective. The retailer cost components will include administrative costs related to obtaining permission for later trading hours and the costs to manage and operate premises during these later hours, including staff salaries, venue maintenance and other associated costs. Due to a lack of data, we are unable to estimate costs for all venues from the retailer perspective. However, evidence from the qualitative studies (two qualitative studies were designed within the ELEPHANT study to understand the policy changes at system level and at venue level) and published sources will be used.

#### Consequences

The economic evaluation will incorporate a number of multisectoral consequences (which can also be thought of as costs/cost savings), including not only to the health service but also the judicial system. The outcomes (consequences) of this study were chosen based on the common alcohol-related harms and outcomes considered in the earlier literature to measure the impact of later trading hours. The primary outcome (consequence) will be alcohol-related ambulance call-outs (table 1). We will collect time series data from the Scottish Ambulance Service for recorded ambulance call-outs in Scotland. The available recorded electronic data on ambulance call-outs include the number of call-outs with date and time, age group, gender and council name. Using a previously developed algorithm, we will then determine which recorded ambulance call-outs are alcohol-related.^31^ To precisely evaluate the effect of the two policies regarding later trading hours for bars and clubs in Glasgow and Aberdeen, we will analyse alcohol-related ambulance call-outs that occurred throughout the weekend night-time (ie, from Friday, 20.00 to Saturday, 6.00 and Saturday 20.00 to Sunday 6.00). In addition, we will analyse all weekend night-time ambulance call-outs and reported crimes as secondary consequences (table 1). Anonymised individual data on reported crimes will be obtained from the Police Scotland IT system. We will use the Scottish Government Justice Directorate crime codes to identify crimes likely linked to alcohol consumption. This dataset will contain types of crimes with date and time, location, etc. Due to a lack of available data, we are not able to measure costs for alcohol-related road-traffic accidents, productivity losses due to alcohol-related harms (ie, reduced workforce participation due to sickness, reduced household labour due to sickness, reduced workforce and household labour due to premature mortality), increased profit for retailers due to later trading hours and increased pleasure for consumers due to additional time for social interaction.

Social service sector (third sector) costs include expenses incurred by voluntary organisations engaged in the NTE, such as volunteer training, medical kits and paramedic services. Consumers’ additional costs during the expanded hours include costs on alcohol, food and other items. Findings from qualitative studies and secondary sources, such as the websites of voluntary organisations, will be used to identify, measure and value the costs to the social service sectors. However, due to a lack of available data, we will not be able to measure the additional costs to consumers during the later trading hours.

Different cost components from multiple sectors are also associated with the primary (alcohol-related ambulance call-outs) and secondary outcomes (all ambulance call-outs and reported crimes). Alcohol-related ambulance call-outs are associated with the costs of major alcohol-related healthcare services including alcohol-related hospital admission, alcohol-related emergency services and ambulance service cost. Costs associated with reported crimes relate to three main cost areas: costs in anticipation of crime, costs as a consequence of crime and costs in response to crime.^64^ The costs of ambulance services and alcohol-related healthcare services will be measured using existing national Information Services Division (ISD) Scottish Health Service cost data. The ISD data will provide the unit cost of each alcohol-related ambulance service, as well as the cost of any subsequent health service utilisation. We will collect ambulance call-out data and whether these call-outs conveyed to hospital or not. If conveyed to the hospital, this will incur costs for the A&E department along with the cost of ambulance services. If an ambulance call does not convey to hospital, this will incur only the cost of ambulance services. Further, we will explore existing evidence from the literature on the percentage of alcohol-related ambulance call-outs that necessitate hospitalisation. Otherwise, we will analyse the costs within the A&E department only due to lack of data to link ambulance call-outs with hospitalisation and will incorporate them into the scenarios analysis. The unit cost for the reported crimes will be adopted from published sources, for example, ‘the economic and social costs of crime’.^64^

#### Perspectives

The base-case analysis (CEA) will be conducted from an National Health Service (NHS) perspective.^58^ We will identify, measure and value the cost of ambulance services, emergency services and hospitalisation services to inform decision makers with a particular focus on the health sector (table 1). The later trading hours may not only impact health services, but may increase criminal justice system costs.^35 36^ Hence, in line with the array of health and non-health sectors consequences, the CCA will incorporate health services, the criminal justice system and societal perspectives. In addition, in the sensitivity analysis, retailers’ and third sector organisations’ perspectives will be incorporated into the societal perspective.

### Economic data analysis

#### Analysis of costs and consequences

The economic evaluation will be conducted separately for Glasgow and Aberdeen. First, we will evaluate the impact of policy changes on harms, then we will conduct a CEA, cost analysis and CCA. Regression methods will be used to estimate effectiveness, that is, the impact of policy changes in Aberdeen and Glasgow. We will fit autoregressive integrated moving average (ARIMA) models with the weekly total counts for the primary and secondary consequences to assess the impact of policy changes (table 1). Such models account for autocorrelation and underlying temporal trends, and we will use Akaike information criterion and Bayesian information criterion to determine the best fitting ARIMA models. After a best fitting model is determined, we will then add the intervention variables to measure the effect size (impact of policy changes).

We will assess whether changes in the primary outcome (alcohol-related ambulance call-outs) have been caused by later trading hours. Where fewer or greater numbers of alcohol-related ambulance call-outs are found to have resulted from the policy changes, the unit cost of each resource will be multiplied by the absolute number of fewer or additional outcomes/consequences, for each city, before and after the policy changes.

#### Subgroup analysis

We will also explore impacts on different groups (men/women, age-groups, socioeconomic deprivation, based on the Scottish Index of Multiple Deprivation quintile scores on a scale of 1–5, where 1 is regarded as most deprived areas and 5 is least deprived areas). We will estimate incremental/decreased alcohol-related ambulance call-outs by available subgroups and then compute total cost to determine the economic impact of later trading hours for bars and clubs on different groups.

#### Time horizon

Deciding the time horizon for an economic evaluation is crucial and usually guided by the periods used to measure costs and consequences.^65^ Data for the primary (alcohol-related ambulance call-outs) and secondary consequences (all ambulance call-outs and reported crimes) will be collected from 2015 to 2022 to assess the impact of policy changes. The policy changes began in 2017 in Aberdeen and in 2019 in Glasgow. The most recently available cost data (unit costs) will be collected, expressed in pounds sterling (£), and if necessary, inflated for the different years using the Health Services index. All costs and consequences will be reported within an annual cycle, so there will be no need to apply discounting.

#### Uncertainty and sensitivity analysis

We will incorporate deterministic sensitivity analysis to explore multiple scenarios. Scenario analysis will be undertaken to assess the impact on the costs for different cost components including health services and the criminal justice system. For example, assuming the percentages of alcohol-related ambulance call-outs required hospitalisation if we do not get a reliable estimate from the published literature (different cost scenarios could include the associated costs, if 5%, 10% or 15% of the conveyed alcohol-related ambulance call-outs required hospitalisation). Similarly, we will create cost scenarios for recorded crimes by assuming percentages of recorded crimes incurred anticipation costs (eg, cost of burglar alarm, CCTV) or consequence costs (eg, cost of stolen property or damage) or in response costs (eg, cost of police and justice system). Moreover, we will also integrate findings from two qualitative studies within the ELEPHANT study and will adapt retailer and third-sector organisations’ perspective in the sensitivity analysis.

## Reporting and discussion

For this natural experiment, the consolidated health economic evaluation reporting guidelines will be used for the economic evaluation report.^66^ Any changes to this protocol will be documented in the final report. Summary cost comparison tables will be generated within and between intervention and control cities.

The planned economic evaluation of this natural experiment has a number of limitations. Unlike RCTs, we cannot control the exposure in natural experiments (ie, policy to implement later trading hours). Hence, due to the lack of randomisation, there is a higher risk of bias and confounding effects influencing the results, which can undermine the validity of the findings.^48^ Therefore, analysing data from natural experiments requires sophisticated statistical methods which can be complex and resource-intensive.^48^ We aim to address the challenges caused by non-randomisation using our planned controlled study design, synthetic control and difference-in-difference regression method to convey a reliable assessment of the effect size and economic impact of later trading hours for bars and clubs. Our research is using routine data from multiple sources. Challenges associated with routine data such as poor data quality due to errors during record-keeping (since routine data are typically maintained for the purpose of registration or record keeping rather than research) and missing information may have an impact on our analysis. However, we will maintain rigorous data cleaning procedures to resolve inconsistencies in the data and will use recommended methods to address any missing data.^48^

We are unable to obtain quantitative data from businesses on sales, profit, employment, staff or other costs arising from the changes. However, we will be able to conduct qualitative interviews within ELEPHANT qualitative studies with a limited number of business owners/managers and will write up their perspectives separately and discuss them in the final economic evaluation report. We are unable to obtain reliable data that could be used to quantify the economic value of the consequences of the later trading hours for bars and clubs in the two cities for consumers beyond health and crime outcomes. Thus, consequences such as pleasure, socialisation, connection or positive or negative impacts on well-being or impacts on the following day (including productivity and wellness) as a result of late-night socialising will not be included. Similarly, due to a lack of data, we are unable to incorporate the costs of alcohol-related road traffic accidents and costs associated with productivity losses (see[Fig F1] figure 1 and table 1). There are also advantages to such a complex natural experiment. This study is less likely to face biases associated with primary data collection, such as measurement errors, a low response rate, recall bias and small sample size because it incorporates data from longitudinal secondary sources.^67^ Hence, this economic evaluation of the natural experiment is expected to provide novel and important evidence on the impact of two newly implemented policies and will inform future decisions and legislation on trading hours. The findings of this study will directly inform local licensing practice and policy, as we will work closely with licensing authorities throughout the research process. We will share our findings with licensing boards and forums prior to publication, enabling them to make evidence-based decisions on licensing hours. Additionally, we will prepare policy briefs and infographics to effectively communicate our findings and will engage with key stakeholders, including Alcohol Focus Scotland, licensing authorities and public health organisations across Scotland, England and Wales. As part of the ELEPHANT study, we will develop a version of the Sheffield Alcohol Policy Model (SAPM) for Aberdeen and Glasgow to assess the long-term effects of later trading hours for bars and clubs. The SAPM model will also allow us to estimate the potential impact of such policy changes in other local authorities across Scotland and England, supporting their decision-making processes. Our study will mainly generate evidence on the impact of later trading hours for bars and clubs in Aberdeen and Glasgow. Therefore, before applying our findings to other local authorities or international contexts, it is required to consider contextual differences such as drinking culture, law enforcement practices and healthcare system.

## Supplementary material

10.1136/bmjopen-2024-095241 online supplemental file 1
