# Supplementary material for: Protocol for an economic evaluation alongside a natural experiment to evaluate the impact of later trading hours for bars and clubs in the night-time economy in Scotland: The ELEPHANT study
Source: BMJ Open. 2025 May 14;15(5):e095241. doi: 10.1136/bmjopen-2024-095241 (PMC12083327; doi:10.1136/bmjopen-2024-095241)
Supplement: online supplemental file 1 [file bmjopen-15-5-s001.docx]

**Appendix 1: Interview topics guide for a qualitative study**

# Evaluating Later or Expanded Premises Hours for Alcohol in the Night-Time Economy (ELEPHANT)

| **Note on using this topic guide:**  **Interviews are likely to be conducted with a range of stakeholders such as**: i) Local authority licensing stakeholders (convenors, clerks, forum chairs, LSOs); planning and environmental health officers and night-time economy managers; ii) Owners/managers of licensed premises and other late night business owners; iii) Service managers/team leaders (e.g. in the police/third sector/health). **Therefore, we will explore the following topics with each group in line with their role/ occupation and in line with our research questions as well as being alert to additional themes raised by the interviewees.**   - This topic guide has been designed to be used flexibly. This means that the question wording, order in which issues are covered and the time spent on different topics will vary between interviews. - The Interviewer and interviewee are free to explore any unanticipated but relevant themes that arise. - The interviewer raises related issues not outlined below as they arise in the course of the data collection, providing that to do so raises no ethical issues or risks to participants not already outlined in the Participant Information Sheets. - The infographic and pandemic timeline should be used by the interviewer during the interview - There is no expectation that all prompts are covered for any one question in the list of topics or in every interview, but **all questions in Section 4 should be asked (spend about 30 minutes on this section)** |
| --- |

*REMEMBER TO PRESS RECORD*

1. **Introduction to the study (5 mins)**

- Interviewer introduction; Thank the participant for agreeing to an interview
- Quick study summary and purpose of the interview:

In Aberdeen, from late 2018, over 30 licensed premises that previously closed at or before 1am were granted permission to close later, up to 3am. In Glasgow, from May 2019, 10 nightclubs that previously closed at 3am are now allowed to close at 4am. The aim of the ELEPHANT study is to understand and assess the impact of these later opening hours on alcohol consumption, services and economic costs/benefits in Glasgow and Aberdeen, including for specific groups. The study will also assess the implications for other UK cities if similar changes were introduced. These interviews will explore your views /experiences regarding the impacts of these later opening hours. We will also explore your views on the future of the night-time economy post COVID-19.

- Remind participants that the interview should take no longer than 1 hour. Remind them about audio recording; check their consent form has been received prior to the discussion.
- Remind participants that what is said in the interview will remain confidential.
- Note that we may ask the interviewee to provide relevant information sources and/or documentation related to the interview content – in some cases they may want to direct us to colleagues to follow up with.
- Check to see if interviewee has looked at the infographics in advance to today’s interview, **and whether they have it to hand**
- Check to see if interviewee has the pandemic timeline in front of them.

1. **Background information about the participant (5 mins)**

- Current job title / position held and where based
- Brief summary of role (incl. roles and responsibilities relevant to this study)

How long have you worked in this role

1. **Recollection of/involvement in the local licensing changes – ALL (5 mins)**

***NOTE FOR INTERVIEWER****: Use the* ***timeline*** *as a guide for this section, and ask questions for Glasgow or Aberdeen as appropriate*

- When did you first hear about these local licensing changes? How did you first find out about them? Were you aware of them before they were introduced?
- Were you aware of these changes prior to us contacting you? [*If no, move onto question 4]*
- The timeline for these local licensing changes was as follows…[*briefly outline the timeline for interviewee*] - does that fit with your memory of what happened, when?
- What was your understanding of the rationale for these changes at the time? Do you remember what was said about the rationale at the time, and by whom?
- What was your involvement in the local licensing changes in Glasgow/Aberdeen (if any), or in any other licensing changes, strategies or research pertaining to those which may be relevant to the interview

1. **Views and experiences (where applicable) of extended opening hours (by stakeholder type): (30 mins)**

**FOR LICENSING (CONVENORS, LICENSING BOARD, CLERKS, FORUM CHAIRS, LSOS, LICENSING LAWYERS) AND NIGHT-TIME ECONOMY MANAGERS:**

- What were/are your (initial) views on the changes in opening hours (in 2018/ 2019 as appropriate to Aberdeen or Glasgow)? (What are your views now?)
- What did you expect to be the impact of the changes at the time?
- What are your views on the successes and challenges of the local opening hours changes?
- **(Glasgow only)** I understand that there were some specific conditions premises had to meet before being granted the extra hour – what can you tell me about that? How important was that in the decision-making? How well do you think the conditions have worked/been adhered to?
- **(Aberdeen only)** Were there any conditions premises had to meet before being granted the extra hour – what can you tell me about them? How important was that in the decision-making? How well do you think the conditions have worked/been adhered to?
- Are there aspects of the changes in extended hours that you would recommend to others, or that you would change, in hindsight? What/why?
- What broader changes do you think occur as a result of extended opening hours? (explore views on positive and negative impacts on business, impacts on consumers (including consumption, violence, accidents, injuries, A&E visits, ambulance call outs, police calls, long-term alcohol related harms/norms and wider costs/benefits to society)
- Do you think there are gaps in knowledge about the impact of these changes that it would be useful to examine?
- What is your view on the costs of late night opening hours to the health service? (e.g. ambulance call outs and hospital treatment) and policing of alcohol harms (attending an accident or an alcohol induced fight/brawl)? Who do you think should pay for these costs? The health service/police, business venues (clubs/pubs) or other?

**FOR OWNERS/MANAGERS OF LICENSED PREMISES (VENUES IN ABERDEEN & GLASGOW)**

**We’ll ask specifically about COVID-19 and opening hours later on in the interview. For now, we’d like to focus back to 2018/19 (as appropriate to Aberdeen or Glasgow):**

- When did you first decide to apply for extended opening hours? Why? If you withdrew your application, can you explain a little about why you did this?
- **(Glasgow only)** I understand that there were some specific conditions premises had to meet before being granted the extra hour – what can you tell me about that? How important was that in the decision-making? How well do you think the conditions have worked/been adhered to?
- **(Aberdeen only)** Were there any conditions premises had to meet before being granted the extra hour – what can you tell me about them? How important was that in the decision-making? How well do you think the conditions have worked/been adhered to?
- **If** **you were granted extended opening hours**, did you use them? If so, when? (i.e. on certain days, for special events/freshers week etc) **(Glasgow only)** Are you still using the extended hours at your venue? Can you tell us why? Is there an economic benefit to your use of the extended hours, or are you using them for other reasons?
- **If you made use of the extended opening hours,** what was impact of these changes on your business (Including extra costs incurred compared to additional sales) and venue operation? Were there any benefits? Any downsides? Do you have a view on whether this has been the same/different for other businesses?
- To open during these extended opening hours, did you have to increase the hours of your existing staff? If yes, please describe the extra staff time and associated costs for one extended hours evening. For example, on an average extended-opening hours night, what were the typical extra hours worked by (how many) staff and what was the extra staff cost of this?
- To open during these extended opening hours, did you have to take on any *new* *staff*?
- Did you have to make any other changes to meet licensing conditions attached to the extended hours? Tell us about the costs of these (if you haven’t already).
- Can you think of any other costs incurred to your business due to extended opening hours? If yes, please describe them.
- Overall, do you think extended opening hours have been worthwhile in economic terms (i.e. net profits compared to net costs) for your business? Why?
- **If you haven’t made use of the extended opening hours**, can you say a little about the reasons for this? Are you likely to use them in the future? When, and why?
- In your role, have you observed any changes in alcohol-related harms (violence, accidents, injuries, A&E visits, ambulance/police call-outs) arising from extended opening hours?
- What other changes do you think are related to extended opening hours? (explore views on positive/negative impacts on business, alcohol-related harms/norms, any wider costs and/or benefits to society)
- Do you think that the impact of later opening hours is well understood?
- Much of the evidence we have suggests that later opening hours leads to more assaults, and ambulance call-outs. What do you think of this? If this is the case, who should pay for these services?
- Have your views on the extended opening hours changed in any way over time? If so, how?

**FOR SERVICE MANAGERS/TEAM LEADERS IN STRATEGIC ROLES ( e.g licensing police people, third sector managers, health managers or transport managers)**

- How do late night opening hours impact on your service/your team, if at all? Have there been any particular impacts that you would say are related to the changes in Glasgow/Aberdeen? Have there been any changes made to your service/team as a result? (i.e. staffing, resources). If so, in which particular area/location?
  - In relation to your area of work, what are the perceived benefits of extended opening hours? And in your view, what are the downsides?
  - In your role, have you observed any changes in alcohol-related harms (consumption, drunkenness, violence, accidents, injuries, A&E visits, ambulance call-outs, police calls) arising from extended opening hours?
- What other changes do you think occur when premises are permitted to open later?
  - positive/negative impacts on business, alcohol-related harms/norms, any wider costs and/or benefits to society
- Do you think that the impact of later opening hours is well understood, or are there gaps in our current understanding?
- Much of the evidence we have suggests that later opening hours leads to more assaults, and ambulance call-outs. What do you think of this? If this is the case, who should pay for these services?
- Have your views on the extended opening hours changed in any way over time? If so, how?

**FOR STAKEHOLDERS DEALING WITH THE PUBLIC IN FRONTLINE OR PUBLIC FACING ROLES (i.e. ‘on the beat’ police, A&E staff, street pastors, taxi marshals) and for LATE NIGHT FOOD OUTLET OWNERS**

- How do late night opening hours impact on your service/your team, if at all? Have there been any particular impacts that you would say are related to the changes in Glasgow/Aberdeen? Have there been any changes made to your service/team as a result? (i.e. staffing, resources). If so, in which particular area/location?
  - In relation to your area of work, what are the perceived benefits of extended opening hours? And in your view, what are the downsides?
  - In your role, have you observed any changes in alcohol-related harms (consumption, drunkenness, violence, accidents, injuries, A&E visits, ambulance call-outs, police calls) arising from extended opening hours?
- What other changes do you think occur when premises are permitted to open later?
  - positive/negative impacts on business, alcohol-related harms/norms, any wider costs and/or benefits to society
- Do you think that the impact of later opening hours is well understood, or are there gaps in our current understanding?
- Much of the evidence we have suggests that later opening hours leads to more assaults, and ambulance call-outs. What do you think of this? If this is the case, who should pay for these services?
- Have your views on the extended opening hours changed in any way over time? If so, how?

**FOR PUBLIC HEALTH, ALCOHOL AND DRUG PARTNERSHIPS (ADP), LICENSING STAKEHOLDERS AND NIGHT-TIME ECONOMY MANAGERS and for COMMUNITY PLANNING TEAMS**

- To what extent are extended are opening hours something that are a concern or focus? Why?
- In relation to [public health/ADP/licensing/community planning], what are the perceived benefits of extended opening hours? And in your view, what are the downsides? What is your overall view of these changes?
- What would you expect to be the impact of the local changes on customer behaviour?
  - Do you have any data that could tell us whether there has been any impact on this?
- What other changes do you think may occur as a result of extended opening hours?
  - (explore views on positive/negative impacts on business, alcohol-related harms/norms, any wider costs and/or benefits to society)
- Do you think that the impact of later opening hours is well understood, or are there gaps in our current understanding?
- Much of the evidence we have suggests that later opening hours leads to more assaults, and ambulance call-outs. What do you think of this? If this is the case, who should pay for these services?
- Have your views on the extended opening hours changed in any way over time? If so, how?

1. **ALL: COVID-19 Restrictions on Licensed Premises** (have pandemic timeline to hand) **(10 mins)**

- For each of the following: What are your views on *other* changes in opening hours and other restrictions/amendments to licensing introduced during the Covid-19 pandemic? How did they affect your area of policy/responsibility/work/your business/service in terms of demand/staffing/shift patterns etc.? How did they affect customer behaviour as above?
- Closures of premises during lockdowns (Mar-Jun 2020; Jan-Feb 2021)
- Outdoor openings only – Apr. 2021 (indoor again May 2021)
- Opening premises but with various curfews on opening hours (throughout 2020)
- Opening premises but with ban on indoor alcohol sales (Oct. 2020)
- Relaxation of licensing rules on outdoor trading/takeaway alcohol
- Were you in a position to observe (and if so what’s your sense of) any changes in alcohol-related harms (consumption, drunkenness, violence, accidents, injuries, A&E visits, ambulance call-outs, police calls) arising from these COVID-19-related changes?
- The trading restrictions during Covid-19 applied only to on-licence premises (bars/pubs/clubs) and not to off-licences (shops/supermarkets/online sales). How, in your experience, has that made a difference to different types of alcohol-related harms?

1. **ALL – Night time economy policies post Covid-19 (15 mins)**

- What are the most important considerations, in your view, given your role, in discussions about licensing going forward, now, following these extended hours and all the other changes during COVID-19? What have we learned from all the changes?
- What are the most important considerations, in your view, given your role, for regulation of late-night alcohol sales now? Would you like to see liberalisation of or restrictions in trading hours for any premises or areas or types of premises? How would that work?
- How can the government and local licensing authorities achieve a good balance between benefits and harms when regulating late night bars and clubs? (i.e. the night time economy)
- The infographic that we sent to you in advance of the interview outlines four possible policy options – potential ‘sweet spot policies’ – that could help Government to reduce alcohol harms whilst allowing late night business and the late night economy to recover post-Covid-19:
  - 1. Raising the price of alcohol
    2. Tackling online alcohol sales
    3. Shaping the night time economy
    4. Managing the night time economy and opening hours
- What are your general thoughts on the infographic?
  - Did anything jump out at you?
  - Did anything surprise you?
- Tell me what you think about sweet spot policies on the basis of this infographic?
- Which of the four areas are you most interested to discuss and why? NOTE: this could also be ascertained by asking:
  - Which area are you most knowledgeable about?
  - Which area would you most support?

Focus on this area and explore the following:

- Did you already know anything about the evidence based underpinning this policy option? (outline the ‘what we know’ section briefly, challenge suggestions of ‘no evidence’/lots of evidence if/when applicable)
- How do you think this policy area might work? How easy/difficult would it be to implement? Why do you think so? (outline the ‘how it could work’ section of the infographic)
- Using the ‘to consider’ sections of the infographic – ask opinions about the specific questions posed
- To what extent to you think this policy option might achieve this balance between reducing alcohol harms whilst allowing late night businesses to recover? (Prompt: what difference would it make, if any, to business? To communities? To residents? To alcohol consumption? To alcohol sales? (Venue owners/managers only) To health services? To police? Any other groups?)
- Can you think of any unintended consequences associated with implementation in Scotland? (i.e. for businesses, for public health, for consumers, for the NTE more generally)
- Are you aware of any other policy options which might achieve this balance?

**7. ALL: Final section**

- Any final comments.
- Discuss possible other people to interview and any other relevant information sources and documentation. Could include documents from police, ambulance, transport or other services relating to staffing patterns, service provision or other issues arising from changed opening hours. Any specific relevant business plans, e.g. shift patterns etc. where individuals are willing to share them, on the basis of assured anonymity. Thank the participant for their time and input.
