# Supplementary material for: Protocol for an economic evaluation alongside a natural experiment to evaluate the impact of later trading hours for bars and clubs in the night-time economy in Scotland: The ELEPHANT study
Source: BMJ Open. 2025 May 14;15(5):e095241. doi: 10.1136/bmjopen-2024-095241 (PMC12083327; doi:10.1136/bmjopen-2024-095241)
Supplement: Supplementary file 2 [file bmjopen-2024-095241.reviewer_comments.pdf]

PEER REVIEW HISTORY

BMJ Open publishes all reviews undertaken for accepted manuscripts. Reviewers are asked to complete a checklist review form and are provided with free text boxes to elaborate on their assessment. These free text comments are reproduced below.

ARTICLE DETAILS

Title (Provisional)

Protocol for an economic evaluation alongside a natural experiment to evaluate the impact of later or expanded premises hours for alcohol in the night-time economy in Scotland: The ELEPHANT study

Authors

Sheikh, Nurnabi; Haghpanahan, Houra; Lewsey, Jim; Angus, Colin; Emslie , Carol; Fitzgerald, Niamh; McIntosh, Emma

---

VERSION 1 - REVIEW

**Reviewer**	**1**
**Name**	**Reeves, Penny**
**Affiliation**	**University of Newcastle Hunter Medical Research Institute, Health Research Economics**
**Date**	**16-Dec-2024**
**COI**	**None**

Page 10 Lines 232 - 239

The description of the ICER for the CEA could be made clearer. What is the metric for the expected numerator - incremental change in the number of call outs, will the ICER present the incremental cost per additional call out?

I think the approach to include both CEA of the primary outcome, supplemented by CCA is wise. The method for the CCA could be clearer.

That is, which consequences (positive and negative) do the authors expect to present in natural units and which will be measured, valued and presented as monetised values?

The discussion would benefit from the authors providing commentary about how the findings from this evaluation will be used to influence policy and whether the results are generalisable to other jurisdictions, including overseas, given the characteristics of the Scottish context

---

**Reviewer**	**2**

**Name**	**Green , Colin P.**
**Affiliation**	**Norwegian University of Science and Technology**
**Date**	**23-Dec-2024**
**COI**	**None**

This is a protocol for evaluating the effect of extending opening hours on ambulance call-outs and crime in 2 cities in Scotland. This is an important, policy-relevant, topic where – as highlighted in the paper – more research is needed.

Comment

1. The core approach follows from two extensions, in Glasgow and in Aberdeen. These are subtlety different policy changes, but at their core they involve venues applying for extended opening hours. It is a little unclear in the proposal (page 8), but it seems that the Glasgow extension was more restrictive in the sense that there were more requirements for venues to demonstrate their suitability. The study aims to study the effect of 10 extensions (venues) in Glasgow and 38 in Aberdeen (all that were granted an extension). What was unclear, are the 10 in Glasgow all that were granted? How many applied but were not granted the extension? In general, and as discussed further below, this leads to concerns of selection bias when focusing on estimating the effect of given venues on outcomes.

2. A main outcome focused on is ambulance call-outs. Undoubtedly this is important, but why was this chosen. Is it due to data availability or because it is the main outcome of social interest.

A related question (and this relates to a question below) how are these call-outs assigned to a particular venue? Is this straightforward to do? What if, for example, longer hours led to more ambulance call-outs at other places? The proposal in general does not provide much details on these types of issues of measurement.

3. The study design begins with the use of interrupted time series (ITS). The proposal could be clearer on what are the underlying assumptions required to interpret the results from this type of analysis. In essence one has to assume that the researcher can model the trend the outcome of interest would have followed in the absence of treatment. This seems particular likely to be difficult here due to the selection into treatment that occurs as discussed above. As an example, if extensions are only granted to venues that are deemed suitable, this may be in part on the basis of lower expected problems associated with a specific venue in the future (i.e. the licensing authority believes the venue is a 'good' trend).

4. Perhaps recognizing these issues a 'controlled' ITS is discussed. A key question is how the comparison groups will be chosen. Are they other venues within Glasgow / Aberdeen. If so parallel trends may be a concern due to the reasons above, the trends of the venues may be part of the selection process both in terms of venues applying for the extension and

receiving the extension. A further concern is the potential for spillovers between treated and comparison venues.

5. An alternative approach is to estimate the whole policy effect at a city level. This is set-out on page 11. This potentially can retrieve an Intention to Treat (ITT) estimate of the effect of the change in policy on the outcomes. This mitigates many of the selection problems highlighted above. It is not clear however (page 12) why an ITS would be credible in the absence of comparators who satisfy the parallel trends assumption. The synthetic control design (page 12) is a more promising approach.

It is a little strange to say that data will be collected for all cities except Glasgow and Aberdeen (is it not "in addition to"?)

6. It is not clear how the ARIMA models discussed on page 16 fits with this earlier discussion. Is this the approach to predict the trends in the ITS model?

Minor comments:

1. On page 6 it is stated that the highest concentration of alcohol-related ambulance call outs occurred between 9pm and 1am, while the highest concentration of non-alcohol related call outs occurred between 6pm and 10pm "revealing that alcohol-related ambulance call outs are more prevalent after midnight". I am not debating that statement, but the logic of that argument seems strange.

2. Page 7. "using routinely collected data via natural experiment methodology" needs rewriting for clarity.

3. Page 7. "existing literature on economic evaluation alongside RCTs does not address important methodological requirements for conducting economic evaluations alongside natural experiments". This statement is very unclear and I am not sure what it means. It would be better to explain what specific issues there are with alternative approaches, and how can your proposed methodology address this.

VERSION 1 - AUTHOR RESPONSE

**Response to reviewer's comments**			
**Reviewer#1**		**Author's response**	**Changes to document *(highlighted in yellow)***
**1.1**	Page 10 Lines 232 - 239<br>The description of the ICER for the CEA could be made clearer. What is the metric for the expected numerator - incremental change in the number of call outs, will the ICER present the incremental cost	Thank you for the reviewer's comments and suggestion. The text has been edited regarding the reviewer's concern.<br><br>*"This CEA will also revert to a cost analysis by monetising the alcohol-related ambulance call-outs. The policy*	Page 10: lines 256 to 262

	per additional call out?	*of later trading hours for bars and clubs is considered as a detrimental policy from a public health perspective, meaning the policy is expected to associate with the additional costs along with the expected incremental alcohol-related ambulance call-outs. The results of the CEA will be reported in terms of the incremental cost (additional cost required to implement the policy) and the incremental alcohol-related ambulance call-outs due to the later trading hours, if any. For example, we will state that, the policy change is associated with the XX number of increased alcohol-related ambulance call-outs and an estimated £XX increased costs incurred to implement the policy."*	
**1.2**	I think the approach to include both CEA of the primary outcome, supplemented by CCA is wise. The method for the CCA could be clearer.<br><br>That is, which consequences (positive and negative) do the authors expect to present in natural units and which will be measured, valued and presented as monetised values?	The text has been revised according to the reviewer's concern.<br><br>*"The CCA will assess the negative consequences of later trading hours for bars and clubs by measuring incremental alcohol-related ambulance call-outs and reported crimes in natural units. We will then identify, quantify, and value the associated resource costs across multiple sectors, including health sector, criminal justice system, business sector, and third sector. The health sector will cover ambulance service costs, A&E service costs, and inpatient care costs. The criminal justice system will account for police service costs, justice system costs, and the costs of physical and emotional harm. The retail or business sector costs will include application processing costs, venue operating costs, and staff salaries for the later trading hours. Third sector will include costs associated with the voluntary organizations such as street pastor, taxi marshal, etc. We will report the monetized values of incremental costs corresponding to these negative consequences across sectors. We cannot measure intangible costs/benefits (e.g., consumer pleasure/losses) within the scope of our study. However, qualitative evidence on the retailer benefits (e.g., additional*	Page 11: lines 276-288

		profits/loss) and consumer benefits will be reflected in our discussion."	
**1.3**	The discussion would benefit from the authors providing commentary about how the findings from this evaluation will be used to influence policy and whether the results are generalisable to other jurisdictions, including overseas, given the characteristics of the Scottish context	Thank you for highlighting this important aspect of policy influence and generalisability. We have added additional text to the discussion section regarding the reviewer's concern.<br><br>*"The findings of this study will directly inform local licensing practice and policy, as we will work closely with licensing authorities throughout the research process. We will share our findings with Licensing Boards and Forums prior to publication, enabling them to make evidence-based decisions on licensing hours. Additionally, we will prepare policy briefs and infographics to effectively communicate our findings and will engage with key stakeholders, including Alcohol Focus Scotland, licensing authorities, and public health organizations across Scotland, England, and Wales. As part of the ELEPHANT study, we will develop a version of the Sheffield Alcohol Policy Model (SAPM) for Aberdeen and Glasgow to assess the long-term effects of later trading hours for bars and clubs. The SAPM model will also allow us to estimate the potential impact of such policy changes in other local authorities across Scotland and England, supporting their decision-making processes. Our study will mainly generate evidence on the impact of later trading hours for bars and clubs in Aberdeen and Glasgow. Therefore, before applying our findings to other local authorities or international contexts, it is required to consider contextual differences such as drinking culture, law enforcement practices, and healthcare system. "*	Page 20: lines 528-536<br>Page 21: lines 537-541
**Reviewer #2**		**Author's response**	**Changes to document *(highlighted in green)***
This is a protocol for evaluating the effect of extending opening hours on ambulance call-outs and crime in 2 cities in Scotland. This is an important, policy-relevant, topic where – as highlighted in the paper – more research is needed.			
**2.1**	The core approach follows from two extensions, in Glasgow and in Aberdeen. These are subtlety	Thank you for your concern. Authors agreed with your understanding that the scope of the policy change is higher in	Please see page 8: lines 192-195 &

	different policy changes, but at their core they involve venues applying for extended opening hours. It is a little unclear in the proposal (page 8), but it seems that the Glasgow extension was more restrictive in the sense that there were more requirements for venues to demonstrate their suitability. The study aims to study the effect of 10 extensions (venues) in Glasgow and 38 in Aberdeen (all that were granted an extension). What was unclear, are the 10 in Glasgow all that were granted? How many applied but were not granted the extension? In general, and as discussed further below, this leads to concerns of selection bias when focusing on estimating the effect of given venues on outcomes.	Aberdeen compared to Glasgow, in terms of volume of the premises and extra trading hours permitted. In Glasgow, 10 nightclubs among the 17 applications were permitted for the 4am pilot scheme. The text has been edited following the reviewer's concern.<br><br>*"In Glasgow, as of 12th April 2019, under a scheme planned by the Glasgow Licensing Board (GLB), 10 nightclubs among the 17 applications submitted (among 1,352 on-premises outlets) were permitted to implement a variation in their licence, enabling them to open for an extra hour until 4am for at least 12 months."*<br><br>*"Although both policy changes involve later trading hours for bars and clubs, the scope and level of exposure in Aberdeen, measured by the number of premises and the hours permitted, is greater than in Glasgow."*	211 and Page 9: 212-213
**2.2**	A main outcome focused on is ambulance call-outs. Undoubtedly this is important, but why was this chosen. Is it due to data availability or because it is the main outcome of social interest.<br><br>A related question (and this relates to a question below) how are these call-outs assigned to a particular venue? Is this straightforward to do? What if, for example, longer hours led to more ambulance call-outs at other places? The proposal in general does not provide much details on these types of issues of measurement.	Thank you for bringing up the issue of outcome selection. We considered outcomes, primary outcome- alcohol-related ambulance call-outs and secondary outcomes- reported crimes, and all ambulance call-outs irrespective of alcohol flagged or not. The outcomes of this study were chosen based on the common alcohol-related harms and outcomes considered in the earlier literature to measure the impact of later trading hours. Text has been added regarding the reviewer's concern on outcome selection.<br><br>*"The outcomes (consequences) of this study were chosen based on the common alcohol-related harms and outcomes considered in the earlier literature to measure the impact of later trading hours.*<br><br>We did not assign call-outs with the permitted premises. We considered Aberdeen and Glasgow as our intervention cities (rather than areas within cities where premises are located), where later trading hour policies have been implemented and compared with a control city where such policy is absent. That means, we	Page 15: lines 392-393<br><br>Page 9: lines 216-222

		will estimate policy effects at the city level. However, we added text regarding the reviewer's concern-<br><br>*"This study includes two Scottish cities as intervention sites (the estimated effect will be at city level), where 10 licensed alcohol premises in Glasgow and 38 licenced alcohol premises in Aberdeen were permitted to operate with later trading hours. The outcomes in these intervention cities will be compared with a control city to assess the impact of later trading hours. Control areas will be selected from other Scottish cities where such policies are not in place. Additionally, we will generate a synthetic control for both Aberdeen and Glasgow. The control selection procedure and synthetic control methodology are described below."*	
**2.3**	The study design begins with the use of interrupted time series (ITS). The proposal could be clearer on what are the underlying assumptions required to interpret the results from this type of analysis. In essence one has to assume that the researcher can model the trend the outcome of interest would have followed in the absence of treatment. This seems particular likely to be difficult here due to the selection into treatment that occurs as discussed above. As an example, if extensions are only granted to venues that are deemed suitable, this may be in part on the basis of lower expected problems associated with a specific venue in the future (i.e. the licensing authority believes the venue is a 'good' trend).	Thank you for the reviewer's comment on ITS design. Regarding the reviewer's concern, we have added assumptions of the ITS and discussed with our study design.<br><br>*"A clear differentiation is required between pre-intervention and post-intervention periods for an ITS study. The 'time' (date) of alcohol policy changes will be regarded an interruption in the time series and will define pre-intervention and post-intervention periods. Data will be collected before and after that interruption, and at least three variables such as a time variable, a policy variable to indicate pre-intervention and post-intervention periods, the outcome variable with values for each time points are required for an ITS study. A short-term outcome is also suggested for an ITS study, in our case alcohol-related ambulance callouts and reported crimes are immediate effect of policy changes. The interruptions in Aberdeen occurred gradually following the approval date for the later trading hours of the 38 bars and clubs. ITS is one of the effective research designs to establish causality when RCTs are not practical"*	Page 9: lines 235-237<br>Page 10: lines 238-245

		Authors agreed with the reviewer's assumption that if good practicing premises allowed to open later, we may expect lower effects. However, the effects we considered in our study (alcohol-related ambulance call-outs and reported crimes) are most likely to be occurred at outside the premises. So, even after ensuring good practice requirements for the premises, such policy changes are expected to increase harms, i.e., alcohol-related ambulance call-outs and reported crimes.	
**2.4**	Perhaps recognizing these issues a 'controlled' ITS is discussed. A key question is how the comparison groups will be chosen. Are they other venues within Glasgow / Aberdeen. If so parallel trends may be a concern due to the reasons above, the trends of the venues may be part of the selection process both in terms of venues applying for the extension and receiving the extension. A further concern is the potential for spillovers between treated and comparison venues.	Thank you for the reviewer's concern. We will follow a novel flow-diagram to assess suitability of our potential control candidates, described in the **Comparators section**. Parallel trend assumption check is one of the main and first condition that will apply to this assessment. We considered the two cities, Aberdeen and Glasgow as the intervention sites (so, estimated effect will be at the city level). Similarly, we considered rest of Scottish cities apart from Glasgow and Aberdeen, as the potential control candidates and will assess their suitability as a control city.<br><br>*"This study includes two Scottish cities as intervention sites (the estimated effect will be at city level), where 10 licensed alcohol premises in Glasgow and 38 licenced alcohol premises in Aberdeen were permitted to operate with later trading hours. The outcomes in these intervention cities will be compared with a control city to assess the impact of later trading hours. Control areas will be selected from other Scottish cities where such policies are not in place. Additionally, we will generate a synthetic control for both Aberdeen and Glasgow. The control selection procedure and synthetic control methodology are described below."*<br><br>Within our controlled interrupted time-series study design and considering city as intervention site, we are expecting to overcome the issues related to the spillovers between intervention and control premises.	Page 9: lines 216-223<br><br>Please see the **Comparators section for the control selection methodology** from the pages 12-14

**2.5**	An alternative approach is to estimate the whole policy effect at a city level. This is set-out on page 11. This potentially can retrieve an Intention to Treat (ITT) estimate of the effect of the change in policy on the outcomes. This mitigates many of the selection problems highlighted above. It is not clear however (page 12) why an ITS would be credible in the absence of comparators who satisfy the parallel trends assumption. The synthetic control design (page 12) is a more promising approach.<br><br>It is a little strange to say that data will be collected for all cities except Glasgow and Aberdeen (is it not "in addition to"?)	We will estimate policy effect at the city level. We have added text to mitigate this confusion considering the reviewer's concern.<br><br>*"This study includes two Scottish cities as intervention sites (the estimated effect will be at city level), where 10 licensed alcohol premises in Glasgow and 38 licenced alcohol premises in Aberdeen were permitted to operate with later trading hours. The outcomes in these intervention cities will be compared with a control city to assess the impact of later trading hours. Control areas will be selected from other Scottish cities where such policies are not in place. Additionally, we will generate a synthetic control for both Aberdeen and Glasgow. The control selection procedure and synthetic control methodology are described below."*<br><br>We agreed with the reviewer that controlled ITS will be more credible if we get a suitable control, and synthetic control is a reliable design. In our control selection procedure, we mentioned if we do not get any suitable control then will proceed with an uncontrolled ITS along with the synthetic control.<br><br>*"If none of the potential control candidates satisfy parallel trends assumption, we will proceed with an uncontrolled ITS study design (**Figure 2**). In addition, we will construct a synthetic control based on all potential control candidates since synthetic control does not need to satisfy parallel trends and will proceed with a controlled ITS study design"*<br><br>The text regarding data collection for synthetic control has been edited regarding the reviewer's concern.<br><br>*"To generate weights for the SCM, we will collect data from all Scottish councils/cities in addition to the Glasgow and Aberdeen"*	Page 9: lines 216-223<br><br>Page 13: lines 332-335 & 338-339
**2.6**	It is not clear how the ARIMA models discussed on page 16 fits	Thank you for the reviewer's concern. We will model the difference of	

	with this earlier discussion. Is this the approach to predict the trends in the ITS model?	outcome between intervention and control cities. The difference will be modelled based on the time series modelling technique, autoregressive integrated moving average (ARIMA) to estimate the effect size (impact of later trading hours). Autoregressive (AR) and moving average (MA) components were used to address the residual autocorrelation of the series of the difference.	
**Minor comments**			
**2.7**	On page 6 it is stated that the highest concentration of alcohol-related ambulance call-outs occurred between 9pm and 1am, while the highest concentration of non-alcohol related call outs occurred between 6pm and 10pm "revealing that alcohol-related ambulance call outs are more prevalent after midnight". I am not debating that statement, but the logic of that argument seems strange.	Thank you for pointing the issue. The text has been edited now.<br><br>*"On weekends in Scotland in 2019, the highest concentration of alcohol-related ambulance call-outs occurred between 9pm and 1am, whereas the highest concentration of non-alcohol-related call-outs appeared between 10am and 9pm; revealing that alcohol-related ambulance call-outs are more prevalent after midnight."*	Page 6: lines 136-139
**2.8**	Page 7. "using routinely collected data via natural experiment methodology" needs rewriting for clarity.	The sentence has been edited following the reviewer's comment.<br><br>*"Economic evaluations using natural experiment methodology based on routine data are becoming increasingly popular"*	Page 7: lines 165-167
**2.9**	Page 7. "existing literature on economic evaluation alongside RCTs does not address important methodological requirements for conducting economic evaluations alongside natural experiments". This statement is very unclear and I am not sure what it means. It would be better to explain what specific issues there are with alternative approaches, and how can your proposed methodology address this.	Thank you for the reviewer's concern. The text has been revised regarding the reviewer's concern.<br><br>*"In addition, the existing literature on economic evaluation alongside RCTs does not highlights important methodological aspects for conducting economic evaluations alongside natural experiments, particularly those related to study design. For example, it provides limited guidance on addressing biases and confounding effects raised from non-randomization, selecting appropriate comparators, and managing routine data from multiple sources."*	Page 7: lines 172-176
